# Low Molecular Weight Heparin Treatment Patterns and Outcomes in Cancer Patients with Acute Venous Thromboembolism: A Nationwide Cohort Study in France

**DOI:** 10.3390/cancers15113011

**Published:** 2023-05-31

**Authors:** Laurent Bertoletti, Gaelle Gusto, Nadia Quignot, Artak Khachatryan, Jose Chaves, Audrey Moniot, Ruth Mokgokong, Isabelle Mahé

**Affiliations:** 1 Université Jean Monnet Saint-Étienne, CHU Saint-Étienne, Mines Saint-Etienne, INSERM, SAINBIOSE U1059, CIC 1408, Département of Médecine Vasculaire et Thérapeutique, F-42023 Saint-Etienne, France; 2 Certara France, 75008 Paris, France; 3 Certara UK, London EC2Y 5EB, UK; 4 Pfizer SLU, 28108 Madrid, Spain; 5 Pfizer SAS, Pfizer, 75668 Paris, France; 6 Pfizer Ltd., Tadworth KT20 7NS, UK; 7 Innovations Thérapeutiques en Hémostase, Service de Médecine Interne, APHP, Inserm UMR_S1140, Hôpital Louis Mourier, Université Paris Cité, 75015 Paris, France

**Keywords:** venous thromboembolism, cancer, anticoagulants, bleeding, low molecular weight heparin, medical database

## Abstract

**Simple Summary:**

Venous thromboembolism (VTE) is a common and often fatal complication in cancer patients. The initial treatment for VTE in cancer patients is typically low molecular weight heparins (LMWH), a type of anticoagulant that prevents VTE from recurring. However, information on outcomes of LMWH treatment in cancer patients with VTE have not typically been collected from large national databases, such as the one in France. The aim of this study was to assess VTE recurrence, bleeding (a common side effect of anticoagulant treatment), and all-cause mortality in cancer patients in France who were prescribed LMWH for VTE in 2013–2018. Our results indicate that cancer patients who experience VTE have unmet medical needs due to high rates of mortality, VTE recurrence, and bleeding after LMWH treatment initiation. Our study may help further research on outcomes in cancer patients with VTE who receive the next generation of therapy, direct oral anticoagulants.

**Abstract:**

Patients with cancer have an increased risk of developing venous thromboembolism (VTE) and an increased risk of death from VTE. Until recently, the standard of care for treatment of VTE in cancer patients was low molecular weight heparins (LMWH). To determine treatment patterns and outcomes, we performed an observational study using a nationwide health database. Treatment patterns, rates of bleeding, and VTE recurrence at 6 and 12 months were assessed in cancer patients with VTE in France prescribed LMWH in 2013–2018. Of 31,771 patients administered LMWH (mean age 66.3 years), 51.0% were male, 58.7% had pulmonary embolism, and 70.9% had metastatic disease. At 6 months LMWH persistence was 81.6%, VTE recurrence had occurred in 1256 patients (4.0%) at a crude rate per 100 person-months (PM) of 0.90, and bleeding had occurred in 1124 patients (3.5%) at a crude rate per 100 PM of 0.81. At 12 months, VTE recurrence had occurred in 1546 patients (4.9%) at a crude rate per 100 PM of 0.71 and bleeding had occurred in 1438 patients (4.5%) at a crude rate per 100 PM of 0.66. Overall, VTE-related clinical event rates were high among patients administered LMWH, suggesting an unmet medical need.

## 1. Introduction

Venous thromboembolism (VTE) comprises deep vein thrombosis (DVT), caused by a blood clot in a deep vein (usually in the leg), and pulmonary embolism (PE), caused by a blood clot that embolized to the lungs. VTE commonly occurs in patients with cancer and those undergoing cancer surgery and is a leading cause of death in these patients [1,2]. Patients with cancer have a four- to seven-fold higher risk of having a VTE event than patients without cancer [1]. VTE may even be underreported in cancer patients, as VTE rates based on autopsy are higher than those based on clinical diagnoses [3,4]. Moreover, VTE recurrence is three- to four-fold more frequent in patients with cancer than in people without cancer [1]. Furthermore, the risk of death from VTE is significantly higher in cancer patients with VTE than in non-cancer patients with VTE [3,5]. For cancer patients, the risk of experiencing a VTE event is influenced by patient age, type and stage of cancer, type of cancer treatment, and other comorbidities [6,7]. Typically, the risk of recurrent VTE is highest in the first few months after diagnosis of a malignancy but can persist for years after the initial VTE, especially if the cancer is still active [3,8]. Therefore, collecting data about VTE-related events in the long term is important.

To prevent recurrence, patients with VTE are prescribed anticoagulants such as low molecular weight heparins (LMWH), direct oral anticoagulants (DOACs) including apixaban and rivaroxaban, and vitamin K antagonists (VKAs) [9]. LMWH have been recommended as the standard of care for initial treatment of VTE in cancer patients for nearly 20 years [10,11]. DOACs are now recommended as an alternative treatment for cancer associated VTE [10,12,13]. However, there is limited real-world evidence in France on treatment patterns and outcomes for cancer patients who are administered LMWH for VTE events, making comparisons of VTE associated outcomes with patients administered DOACs difficult. Using data from a large nationwide healthcare database, we assessed long-term (up to 6 months) and extended (beyond 6 months) treatment patterns, as well as rates of VTE recurrence, bleeding, and mortality in cancer patients with VTE in France who were administered LMWH in 2013–2018. The aim was to collect real-world data on the treatment patterns and outcomes of patients with cancer who were treated with LMWH after a VTE.

## 2. Methods

### 2.1. Overall Study Design and Data Source

The study design and data source have been reported in a previous publication [14]. Briefly, this was an observational study using data from the French national health data system (Système National des Données de Santé [SNDS]), which covers approximately 99% of the French population (EU PAS registration number: EUPAS35888).

### 2.2. Study Population

The analysis included adult (≥18 years) patients with active cancer who were hospitalized for a VTE between 1 January 2013 and 30 June 2018 [14] and who were reimbursed for LMWH (the index treatment) within 30 days after hospital discharge. The study included patients who were hospitalized for reasons other than VTE, but VTE had to be the primary discharge diagnosis. The International Classification of Diseases 10th Revision (ICD-10) codes used to identify patients with a VTE diagnosis were previously reported [14]. Active cancer was defined as a cancer diagnosis or cancer treatment within 6 months before or 30 days after the first VTE diagnosis during the study period (the index VTE diagnosis is the first recorded VTE event and the index VTE event date is the date of the first admission for VTE hospitalization) [14]. Patients who were anticoagulant naïve (AC-naïve) or who had previously received anticoagulant treatment (AC-experienced) were included. AC-naïve patients had no evidence of oral or parenteral anticoagulant use during the 24 months preceding the index VTE diagnosis, unless such therapy was administered prophylactically (only for hip or knee replacements). AC-experienced was defined as evidence of non-prophylactic oral or parenteral anticoagulant use during the 24 months preceding the index VTE diagnosis. Exclusion criteria are detailed in a previous publication [14].

### 2.3. Study Outcomes

The study outcomes chosen for analysis were included because of their clinical relevance. Rates of bleeding leading to hospitalization, recurrent VTE, all-cause mortality, and chronic thromboembolic pulmonary hypertension (CTEPH) were assessed at 6 and 12 months after the index date; the index date is defined as the date of the first recorded AC reimbursement occurring within 30 days after the index VTE event. CTEPH was included as an outcome in the study because it is a potentially life-threatening long-term complication of VTE, including in cancer patients [15], as a result of the risks of cardiotoxicity from cancer treatment [16].

Treatment persistence, discontinuation, and switching were analyzed. Persistence was defined as no discontinuation, interruption, or switching of LMWH treatment during the follow-up period. Discontinuation was defined as no evidence of LMWH reimbursement for at least 30 days from the estimated end of supply of LMWH treatment. Interruption was defined as a gap with no new treatment for less than 30 days after the estimated end of supply of LMWH, with subsequent restart of LMWH treatment after this period. Switching was defined as prescription of a class of anticoagulant other than LMWH at least 1 day after the last reimbursement date for LMWH and within 30 days after the estimated end of supply of LMWH.

### 2.4. Statistical Analysis

Descriptive statistics were calculated for patient demographics, clinical characteristics, and study outcomes. Data for AC-experienced and AC-naïve patients were compared by chi-square test (categorical variables) or ANOVA (continuous variables). The follow-up period ran from the day after the index date to the date of death, loss to follow-up, or end of study (whichever was earliest) and patient data were censored if LMWH treatment was switched, discontinued, or interrupted, or if the patient died. Treatment patterns were analyzed in the timeframes 0–3 months, >3–6 months, >6–12 months, and >12 months. Outcome rates were evaluated at 6 months and 12 months. To ensure anonymization and to comply with data privacy requirements, exact numbers and percentages were not reported when the number of patients was <10.

In a sensitivity analysis, outcomes were analyzed separately for AC-naïve and AC-experienced cohorts.

In an exploratory analysis, patients who remained on treatment were separated into subgroups based on the most common treatment scenarios after VTE in clinical practice. Subgroup 1 included patients who remained on LMWH throughout the study period. Subgroup 2 included patients who were prescribed LMWH as their index treatment and who later switched to an oral anticoagulant (OAC).

## 3. Results

### 3.1. Patient Selection and Characteristics

**Figure 1:**
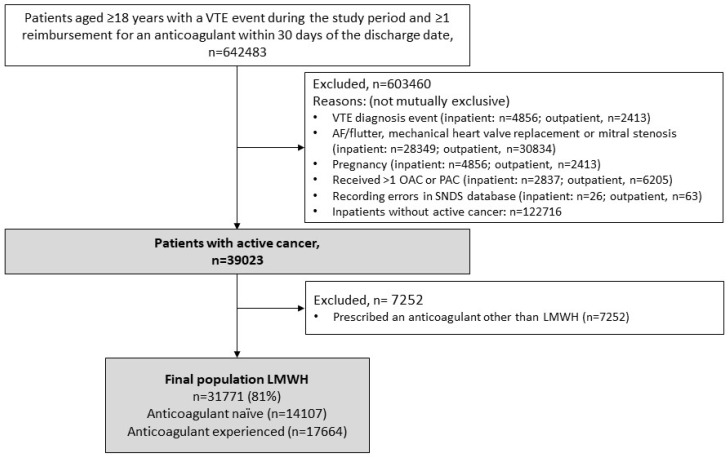
Selection of the study population. LMWH, low molecular weight heparin; VTE, venous thromboembolism.

**Figure 2:**
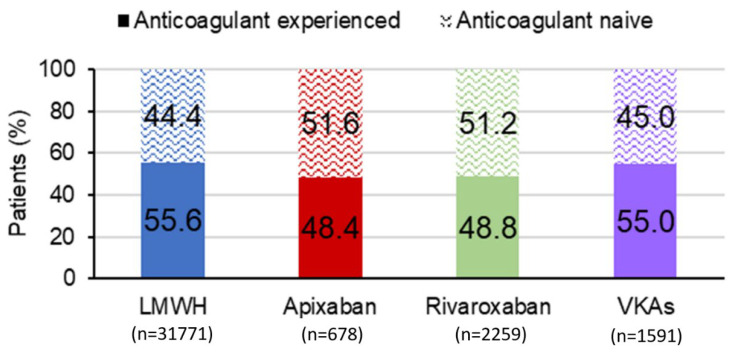
Total numbers of patients with VTE and active cancer prescribed anticoagulants LMWH, low-molecular weight heparin; VKA, vitamin K antagonists.

In the study period, the majority of patients with VTE and active cancer (n = 39,023) received LMWH as their first anticoagulant (n = 31,771, 81.4%); only 678 (1.7%) were treated with apixaban, 2259 (5.8%) with rivaroxaban, and 1591 (4.1%) with VKAs (Figure 1 and Figure 2, and Table 1). The remaining 2724 patients received unfractionated heparin or fondaparinux.

Out of 31,771 patients prescribed LMWH, 14,107 (44.4%) were anticoagulant naïve and 17,664 (55.6%) were anticoagulant experienced. Overall, mean (standard deviation [SD]) age was 66.3 (13.2) years and about half of patients (51.0%) were male. The number of patients with active cancer who initiated LMWH treatment was similar across index years, although there were fewer patients in 2016. Most patients (69.2%) had >4 comorbidities according to the Charlson Comorbidity Index. About a third of patients (33.9%) were prescribed non-steroidal anti-inflammatory drugs (NSAIDs) during follow-up, while 18.4% were prescribed antiplatelets and 2.7% were prescribed hormone therapy. Overall, most patients (58.7%) had PE (with or without DVT); (Table 2). Based on cancer types, most patients had a high (45.2%) or very high (15.6%) risk of VTE (as assessed using the Khorana score [17]). Cancer types with a high risk for VTE included lung (24.9%), lymphoma (5.6%), gynecologic (10.0%), bladder (6.0%), testicular (0.9%), and renal cell carcinoma (3.9%). Cancer types with a very high risk for VTE included brain (3.6%), pancreatic (8.0%), and stomach (4.3%). Other cancer types included breast (13.8%), prostate (8.4%), and colorectal (16.9%). Most patients (70.9%) had metastatic disease and 30.7% of those with metastatic disease had primary lung cancer. Information on cancer treatments received by LMWH-treated patients is shown in Table S1.

### 3.2. Treatment Patterns

Among VTE patients with active cancer who were still alive at 3 months (n = 24,944), 6758 (27.1%) had stopped (discontinuation and/or interruption) or switched their index treatment (Table S2). Among VTE patients with active cancer who were still alive at 12 months (n = 16,612) 3959 (23.8%) had stopped or switched their index treatment. Among patients who were still alive (n = 20,882), persistence of LMWH use was high (n = 17,033, 81.6%) at 6 months after the index VTE diagnosis (Table 3). When treatment switching occurred (n = 2645), it was mainly to rivaroxaban (n = 936, 35.4%), VKAs (n = 561, 21.2%), or apixaban (n = 435, 16.4%).

### 3.3. Clinical Outcomes at 6 Months

In the overall cohort, VTE recurrence occurred in 1256 patients (4.0%) at a crude rate per 100 person-months (PM) of 0.90 (95% confidence interval [CI] 0.86–0.95) (Table 4 and Figure S1). Bleeding leading to hospitalization occurred in 1124 patients (3.5%) overall. The crude rate of bleeding leading to hospitalization per 100 PM was 0.81 (95% CI 0.76–0.86) in the overall cohort. Gastrointestinal (GI) bleeding occurred in 432 patients (1.4%) at a crude rate per 100 PM of 0.31 (95% CI 0.28–0.34) and was the most common type of bleeding. Overall, CTEPH occurred in 58 patients (0.2%). CTEPH occurred at a crude rate per 100 PM of 0.04 (95% CI 0.03–0.05) in the overall cohort. All-cause death occurred in 10,383 patients (32.7%) overall at a crude rate per 100 PM of 7.4 (95% CI 7.3–7.6). Outcomes were consistent between AC-naïve and AC-experienced patients.

### 3.4. Clinical Outcomes at 12 Months

VTE recurrence occurred in 1546 patients (4.9%) in the overall cohort (Table 5). The crude rate of VTE recurrence per 100 PM was 0.71 (95% CI 0.67–0.74) in the overall cohort. Bleeding leading to hospitalization occurred in 1438 patients (4.5%) in the overall cohort at a crude rate per 100 PM of 0.66 (95% CI 0.62–0.69). GI bleeding occurred in 550 patients (1.7%) at a crude rate per 100 PM of 0.25 (95% CI 0.23–0.27) and, as at 6 months, was the most common type of bleeding. CTEPH occurred in 74 patients (0.2%) overall. In the overall cohort, all-cause death occurred in 14,124 patients (44.5%) at a crude rate per 100 PM of 6.44 (95% CI 6.34–6.55). Again, outcomes were consistent between AC-naïve and AC-experienced patients.

### 3.5. Patient Subgroups

Two notable subgroups of patients were identified; 2710 patients were not included in either of these subgroups due to LMWH discontinuation, treatment interruption, or switching to a different anticoagulant administered intravenously. Subgroup 1 (n = 25,256) included patients with persistent use of LMWH and subgroup 2 (n = 3805) included patients who switched from LMWH to OACs (Table S3). Mean age (SD) and proportion of males were similar between subgroups: 66.1 (13.2) years and 51.5% males in subgroup 1 and 67.2 (12.9) years and 48.6% males in subgroup 2. Over half of patients in subgroup 1 (57.5%) had PE (with or without DVT) and most (74.9%) had metastatic disease, whereas in subgroup 2, just over two-thirds of patients (68.0%) had PE (with or without DVT) and about half (52.8%) had metastatic disease.

### 3.6. Outcomes at 6 Months in the Patient Subgroups

VTE recurrence occurred in 1038 patients (4.1%) in subgroup 1 at a crude rate per 100 PM of 0.90 (95% 0.85–0.96) and in 131 patients (3.4%) in subgroup 2 at a crude rate per 100 PM of 0.85 (95% CI 0.72–1.01) (Table S4). In subgroup 1, 1008 patients (4.0%) had bleeding, at a crude rate per 100 PM of 0.87 (95% CI 0.82–0.93); in subgroup 2, 66 patients (1.7%) had bleeding, at a crude rate per 100 PM of 0.43 (95% CI 0.34–0.55). The proportion of patients who experienced CTEPH was 0.2% in subgroup 1 (n = 48); <10 patients in subgroup 2 experienced CTEPH. CTEPH occurred at a crude rate per 100 PM of 0.04 (95% CI 0.03–0.06) in subgroup 1 and 0.05 (95% CI 0.02–0.10) in subgroup 2. All-cause death occurred in 10,383 patients in subgroup 1 (41.1%) at a crude rate per 100 PM of 9.01 (95% CI 8.84–9.17). All-cause death was not calculated for patients in subgroup 2 because the data were censored after treatment switching.

### 3.7. Outcomes at 12 Months in the Patient Subgroups

VTE recurrence occurred in 1264 patients (5.0%) in subgroup 1 at a crude rate per 100 PM of 0.68 (95% CI 0.64–0.71) and in 178 patients (4.7%) in subgroup 2 at a crude rate per 100 PM of 0.84 (95% CI 0.73–0.98) (Table S5). In subgroup 1, 1301 patients (5.2%) had bleeding, at a crude rate per 100 PM of 0.70 (95% CI 0.66–0.74); in subgroup 2, 81 patients (2.1%) had bleeding, at a crude rate per 100 PM of 0.38 (95% CI 0.31–0.48). The proportion of patients who experienced CTEPH was 0.2% in subgroup 1 (n = 60); <10 patients in subgroup 2 experienced CTEPH. CTEPH occurred at a crude rate per 100 PM of 0.03 (95% CI 0.02–0.04) in subgroup 1 and 0.04 (95% CI 0.02–0.08) in subgroup 2. All-cause death occurred in 14,124 patients (55.9%) in subgroup 1 at a crude rate per 100 PM of 7.56 (95% 7.44–7.68). All-cause death was not calculated for patients in subgroup 2 because the data were censored after treatment switching.

## 4. Discussion

A recent study indicated that about one in two patients seen in clinical practice for acute cancer-associated thrombosis would not be eligible for a DOAC randomized, controlled trial [18]. For some of these patients (e.g., those with severe renal failure), LMWH remains the main option, supporting the need for real-world data such as that provided in the current study. Most cancer patients diagnosed with VTE in France were treated with LMWH during the study period, in accordance with the treatment guidelines at the time of the study. VTE-related clinical event rates were high in cancer patients treated with LMWH, most of whom had metastatic disease and aggressive cancer types, suggesting a key unmet medical need. Additionally, mortality was very high at both 6 and 12 months. The high comorbidity burden in the patients included in our study may partially explain the high clinical event rates.

The high clinical event rates in our study are also highlighted when results are compared to the same outcomes in patients without cancer. In an observational real-world database study in France that used the same methodology and study time periods, patients without cancer who had a VTE event experienced lower incidence rates of bleeding, all-cause death, and VTE recurrence after treatment with rivaroxaban, apixaban, and VKA than patients in the current study [14]. For instance, at 6 months after the initial VTE event, the incidence of bleeding that required hospitalization ranged from 0.15–0.43 per 100 PM (calculated from PY) in non-cancer patients whereas it was 0.81 per 100 PM in patients with cancer. All-cause death incidence in non-cancer patients ranged from 0.23–1.02 per 100 PM, whereas it was 7.47 per 100 PM in patients with cancer. VTE recurrence ranged from 0.28–0.42 per 100 PM in non-cancer patients vs. 0.90 per 100 PM in patients with cancer.

The crude VTE recurrence and overall bleeding rates at 6 and 12 months show some similarities to and differences from what was found in other studies. For example, in PREDICARE, a prospective observational study of patients with cancer and VTE prescribed tinzaparin in France, the incidence of VTE recurrence at 6 months was higher (7.3%) than in our study (4.0%) [19]. This difference in incidence rate may have been due to differences in study design and the smaller patient population in PREDICARE (409 patients). Additionally, because our study included patients who had a VTE event triggered by cancer surgery, the prognosis of those patients was likely better than the patients in the PREDICARE study. In the prospective single arm interventional TiCAT (Tinzaparin in Cancer-Associated Thrombosis) study, VTE recurrence at 1–6 months (4.5%) and 1–12 months (5.3%) was similar to VTE recurrence at 6 months and 12 months in our study [20]. Our results for VTE recurrence incidence rate per 100 person-years are also similar to a long-term observational cohort study in the UK in cancer patients who had VTE [21]. The mean crude recurrent VTE incident rate per 100 person-years over the course of that study was 9.6 (0.80 per 100 PM) [21]; our combined PE and DVT incident rate per 100 person-years was 10.8 (0.90 per 100 PM) at 6 months and 8.5 (0.71 per 100 PM) at 12 months. In the TiCAT study, the rate of clinically relevant bleeding (major and non-major) after an initial VTE event was similar at 1–12 months (0.8% per PM) as at 1–6 months (0.9% per PM) [20], which was different from our study, in which the crude rate of overall bleeding leading to hospitalization was lower at 12 months (0.66 per 100 PM) than at 6 months (0.81 per 100 PM). The differences between our findings and those of the TiCAT study could be due to TiCAT being an interventional study with a defined patient sample, whereas our study was a retrospective analysis of a national cohort that included a wider spectrum of patients with cancer. Additionally, the TiCAT study included clinically relevant non-major bleeding, which typically does not lead to hospitalization and may also explain why the bleeding rates in our study were lower. In a retrospective non-interventional study in France showed that patients with cancer treated with the LMWH drug tinzaparin and followed up for 6–12 months after an initial VTE event had similar rates of VTE recurrence (5.7%) and clinically relevant bleeding (5.1%) as in our study (4.0% at 6 months and 4.9% at 12 months for VTE recurrence, 3.5% at 6 months and 4.5% at 12 months for overall bleeding leading to hospitalization) [22].

CTEPH occurred in a small proportion of our study patients. Cancer has been shown to increase the risk of CTEPH [23]. However, limited data exists on the proportion of cancer patients with VTE who go on to develop CTEPH. Despite possible under-reporting, our results indicate that risk of CTEPH in cancer patients with VTE is low. Our results may serve as an epidemiologic reference for future studies. Additionally, the European Society of Cardiology Working Group on Pulmonary Circulation and Right Ventricular Function recommends regular screening for CTEPH in patients after an initial PE event [24], including in patients with cancer as its treatment can cause cardiotoxicity [16]. If regular screening for CTEPH is incorporated into clinical practice, more accurate numbers of patients with CTEPH will likely become available.

We found that recurrent VTE, bleeding, and mortality were frequent in patients with cancer prescribed LMWH. Although LMWH as a monotherapy in cancer patients has long been supported by the American Society of Clinical Oncology, the National Cancer Comprehensive Network, the American Society of Hematology, and the American College of Chest Physicians, more recent guidelines also recommend DOACs as an alternative to LMWH in the first 6 months of treatment [10,12,13,25]. A recent meta-analysis reported increased efficacy (a significantly reduced frequency of VTE recurrence) with DOACs vs. LMWH in cancer patients with VTE [26], indicating that DOACs may be preferred over LMWH in cancer patients to reduce the risk of VTE recurrence.

Data on the bleeding risk with DOACs vs. LMWH is conflicting. In the meta-analysis mentioned above, the risk of major bleeding was not different between DOACs and LMWH, but the risk of clinically relevant bleeding (major and non-major) was significantly higher with DOACs than with LMWH [26]. Considering individual DOACs, apixaban was not associated with an increased risk of major bleeding (mainly GI bleeding) in ADAM VTE [27] or CARAVAGGIO [28], a finding confirmed by a real-world evidence study [29]. However, other DOACS were reported to result in more bleeding than with LMWH, including in the GI tract [30,31]. Clinical trials have also confirmed that GI cancers are more likely to bleed than other types of cancer [32]. To support the clinical trial findings, updated guidelines for DOAC treatments in patients with GI cancers have been published [12,33,34]. It is also important to note that GI bleeding was also the most common type of bleeding in the patients in our study. In the clinic, it is important to be able to identify patients who are at risk for GI bleeding so that the safest AC medications can be prescribed to them. Because of the increased risk of GI bleeding after AC therapy, a predictive score for identifying GI bleeding risk was recently published [35] and may help clinicians determine which AC therapy to prescribe.

We found that the rates of VTE recurrence (4.0–4.9%) and bleeding leading to hospitalization (3.5–4.5%) were similar at 6 and 12 months. These results highlight the challenge of managing VTE in patients with cancer, especially beyond 6 months of treatment as previously described in the literature [36]. Individualized and tailored treatment plans may be beneficial for extended treatment periods after initial VTE. Here, the results of ongoing clinical trials may help clinicians in selecting optimal doses for patients. For instance, the EVE and API-CAT trials are exploring which dose of apixaban is the most appropriate or has the most favorable benefit to risk ratio for extended treatment in patients with cancer-associated VTE [37,38].

A strength of our study is that the SNDS is a large nationwide observational database, which allowed us to identify a cohort of thousands of cancer patients prescribed anticoagulants for VTE. The SNDS covers about 99% of the French population and therefore includes patients with a wide sociodemographic range. Additionally, with such a large population included in the database, we were able to estimate event rates more accurately, especially for rare outcomes such as CTEPH. Another strength of our study is that we collected follow-up data until 12 months after the index VTE diagnosis. Because patients with cancer and VTE have poor survival rates, there is limited published data beyond 6 or 9 months, with only a few studies reporting outcomes and treatment patterns at 12 months [20,22,30]. A limitation is that anticoagulant use was assessed only by reimbursement after hospital discharge. Moreover, patient adherence to prescribed treatment is unknown. Another limitation is that a competing risk analysis for cancer-specific mortality vs. other causes for mortality was not conducted; however, we acknowledged the competing risk by stratifying the presentation of data according to different time points and included a carefully selected choice of denominators (among alive patients, etc.). A further limitation of the study is that some data were missing, including for patients who did not return home after being discharged from hospital but were instead transferred to rehabilitation centers where drugs are dispensed outside of the reimbursement system.

Cancer management (chemotherapy, surgery, radiotherapy) may affect the risks of events such as recurrent VTE and bleeding. Unfortunately, our data, based on a national medico-administrative database, did not allow us to fully describe individual events, as would have been possible with a prospective cohort with an independent adjudication committee. Additionally, our study focused on data from patients hospitalized with VTE because diagnosis codes are not available for non-hospitalized patients, which may have led to underestimated VTE-related events. However, most patients undergoing anticoagulant therapy to prevent VTE recurrence are typically managed in the hospital and not in other healthcare settings. Moreover, it is possible some of the patients in our study were receiving palliative care at the end of life, leading to possible underreporting of VTE related events [39]. However, we expect the number of missing events due to palliative care to be very low because in France, most palliative care facilities are part of the hospital system and the events that occurred in palliative care would have been included in the database used in our study. Lastly, our study included a relatively large percentage of patients who were already AC-experienced (55.6%); this may have been due to previous AC treatment after surgical procedures related to cancer treatment (thromboprophylaxis).

Most patients who switched to an OAC were prescribed rivaroxaban or apixaban, neither of which were recommended by guidelines in France at the time this study was conducted. We hypothesized that patients who switched from LMWH to an OAC (subgroup 2) would have less severe disease than those who continued on LMWH (subgroup 1). Our reasoning was that if clinicians would risk switching patients to a non-recommended anticoagulant, this was likely because these patients had less severe disease than patients who continued on LMWH. Our findings support this reasoning: patients in subgroup 2 had lower percentages of very high- or high-risk cancers and metastatic disease and fewer comorbidities than those in subgroup 1. Additional work is planned to identify determinants of treatment switching and treatment choices after VTE recurrence or bleeding.

## 5. Conclusions

This study may be a steppingstone for future comparisons of LMWH to DOACs in cancer patients with VTE using real-world data. LMWH has been the standard of care for patients with cancer-associated VTE, yet rates of recurrent VTE, bleeding, and mortality remain high in this patient population. DOACs offer a potentially safer and more effective alternative to LMWH in cancer patients with VTE. More real-world data is needed to determine the relative effectiveness and safety of DOACs and LMWH which will help clinicians to offer a more tailored treatment approach for each patient.

## Figures and Tables

**Table 1:** Demographics, index year, and comorbidities.

	LMWH			
	All (n = 31,771)	AC-Naïve (n = 14,107)	AC-Experienced (n = 17,664)	*p*-Value (AC-Experienced vs. AC-Naïve) ^a^
Age at index date (years), mean (SD)	66.3 (13.2)	65.1 (13.5)	67.2 (12.9)	<0.0001
Male, *n* (%)	16,189 (51.0)	6761 (47.9)	9428 (53.4)	<0.0001
Index year, *n* (%)				
2013	5582 (17.6)	2582 (18.3)	3000 (17.0)	0.005
2014	6081 (19.1)	2682 (19.0)	3399 (19.2)	
2015	6220 (19.6)	2775 (19.7)	3445 (19.5)	
2016	4552 (14.3)	1987 (14.1)	2565 (14.5)	
2017	6201 (19.5)	2656 (18.8)	3545 (20.1)	
2018 (January to June only)	3135 (9.9)	1425 (10.1)	1710 (9.7)	
Deprivation index Q4, *n* (%)	11,385 (35.8)	4990 (35.4)	6395 (36.2)	0.12
Deprivation index Q5, *n* (%)	1657 (5.2)	779 (5.5)	878 (5.0)	0.028
Baseline comorbidities (in the 24 months prior to the index date)				
CCI, mean (SD)	6.5 (3.2)	6.3 (3.2)	6.5 (3.2)	<0.0001
CCI 2, *n* (%)	6647 (20.9)	3210 (22.8)	3437 (19.5)	<0.0001
CCI 3, *n* (%)	3130 (9.9)	1382 (9.8)	1748 (9.9)	
CCI 4 or more, n (%)	21,994 (69.2)	9515 (67.4)	12,479 (70.6)	
Baseline bleeding, all diagnosis, *n* (%)	4006 (12.6)	1561 (11.1)	2445 (13.8)	<0.0001
Baseline bleeding, principal diagnosis only, *n* (%)	1305 (4.1)	509 (3.6)	796 (4.5)	<0.0001
Comorbidities, *n* (%)				
Hypertension	11,343 (35.7)	4694 (33.3)	6649 (37.6)	<0.0001
Anemia	9619 (30.3)	3992 (28.3)	5627 (31.9)	<0.0001
Diabetes	5213 (16.4)	2046 (14.5)	3167 (17.9)	<0.0001
Pulmonary disease	3731 (11.7)	1225 (8.7)	2281 (12.9)	<0.0001
Obesity	3724 (11.7)	1582 (11.2)	2142 (12.1)	0.012
Cerebrovascular disease	1452 (4.6)	567 (4.0)	885 (5.0)	<0.0001
Moderate to severe renal disease	1421 (4.5)	534 (3.8)	887 (5.0)	<0.0001
History of falls	649 (2.0)	278 (2.0)	371 (2.1)	0.42
Concomitant treatment during entire follow-up, *n* (%)				
Antiplatelets	5832 (18.4)	2506 (17.8)	3326 (18.8)	0.015
NSAIDS	10,767 (33.9)	4951 (35.1)	5816 (32.9)	<0.0001
Hormone therapy ^b^	844 (2.7)	447 (3.2)	397 (2.2)	<0.0001

^a^ Chi-square test for categorical variables and ANOVA for continuous variables. ^b^ ATC codes for estrogens and progesterone: G02BA03, G02BB01, G03A, G03C, G03F. Abbreviations: AC, anticoagulant; ATC, Anatomical Therapeutic Chemical; CCI, Charlson Comorbidity Index; LMWH, low molecular weight heparin; NSAIDS, non-steroidal anti-inflammatory drugs; Q, quintile; SD, standard deviation.

**Table 2:** Types of VTE and cancer.

	LMWH			
Characteristics	All (n = 31,771)	AC-Naïve (n = 14,107)	AC-Experienced (n = 17,664)	*p*-Value (AC-Experienced vs. AC-Naïve) ^a^
Type of VTE, *n* (%)				
DVT without PE	13,109 (41.3)	5836 (41.4)	7273 (41.2)	0.73
PE (with or without DVT)	18,662 (58.7)	8271 (58.6)	10,391 (58.8)	
Risk of incident VTE based on cancer type, *n* (%) ^b^				
Very high-risk cancer types	4941 (15.6)	1444 (10.2)	3497 (19.8)	<0.0001
Pancreatic	2541 (8.0)	741 (5.3)	1800 (10.2)	<0.0001
Stomach	1359 (4.3)	390 (2.8)	969 (5.5)	<0.0001
Brain	1149 (3.6)	343 (2.4)	806 (4.6)	<0.0001
High-risk cancer types	14,359 (45.2)	7439 (52.7)	6920 (39.2)	<0.0001
Lung	7899 (24.9)	4261 (30.2)	3638 (20.6)	<0.0001
Gynecologic ^c^	3191 (10.0)	1466 (10.4)	1725 (9.8)	<0.0001
Bladder	1915 (6.0)	601 (4.3)	1314 (7.4)	<0.0001
Lymphoma	1791 (5.6)	1023 (7.3)	768 (4.4)	<0.0001
Renal cell carcinoma	1240 (3.9)	708 (5.0)	532 (3.0)	<0.0001
Testicular	296 (0.9)	175 (1.2)	121 (0.7)	<0.0001
Other cancer types	11,928 (37.5)	4903 (34.8)	7025 (39.8)	<0.0001
Colorectal	5358 (16.9)	1754 (12.4)	3604 (20.4)	<0.0001
Breast	4381 (13.8)	2486 (17.6)	1895 (10.7)	<0.0001
Prostate	2666 (8.4)	1004 (7.1)	1662 (9.4)	<0.0001
Unknown cancer (ICD-10 information missing)	543 (1.7)	321 (2.3)	222 (1.3)	<0.0001
Metastatic vs. non metastatic disease, *n* (%)				
Metastatic disease (ICD-10: C77-C80)	21,994 (70.9)	9637 (69.8)	12,357 (71.8)	<0.0001
Non-metastatic disease	8492 (27.4)	3857 (27.9)	4635 (26.9)	
Unknown (ICD-10 information missing)	543 (1.7)	321 (2.3)	222 (1.3)	
Primary lung cancer ^d^	6749 (30.7)	3626 (37.6)	3123 (25.3)	<0.0001
Reimbursement for cancer therapy in the 24 months before the index date, *n* (%)				
Reimbursement for immunotherapy	849 (2.7)	425 (3.0)	424 (2.4)	0.0008
Reimbursement for hormonotherapy for cancer ^e^	21 (0.1)	12 (0.1)	<10	0.24
Reimbursement for radiation	5319 (16.7)	2527 (17.9)	2792 (15.8)	<0.0001
Reimbursement for chemotherapy	23,554 (74.1)	10,878 (77.1)	12,676 (71.8)	<0.0001

^a^ Chi-square test for categorical variables and ANOVA for continuous variables. ^b^ Based on the Khorana score. ^c^ Gynecologic cancers included malignant neoplasms of the vulva, vagina, cervix uteri, corpus uteri, uterus part unspecified, ovary, placenta, and other and unspecified female genital organs. ^d^ Number of patients with lung cancer including those with metastases. ^e^ Endocrine therapy used specifically in the treatment of neoplastic diseases: ATC code L02. Abbreviations: AC, anticoagulant; ATC, Anatomical Therapeutic Chemical; DVT, deep vein thrombosis; ICD, International Classification of Diseases; LMWH, low molecular weight heparin; PE, pulmonary embolism; VTE, venous thromboembolism.

**Table 3:** Treatment patterns in patients who remained alive at 6 months.

	LMWH			
	All (n = 20,882)	AC-Naïve (n = 9442)	AC-Experienced (n = 11,440)	*p*-Value (AC-Experienced vs. AC-Naïve) ^a^
Treatment patterns, 6 months, *n* (%)				
Treatment discontinuation	307 (1.5)	151 (1.6)	156 (1.4)	0.23
Treatment interruption	897 (4.3)	436 (4.6)	461 (4.0)	0.08
Treatment persistence	17,033 (81.6)	7603 (80.5)	9430 (82.4)	0.0017
Switching	2645 (12.7)	1252 (13.3)	1393 (12.2)	0.07
Time to switching (days), mean (SD)	80.7 (51.8)	82.15 (52.6)	79.4 (51.1)	0.06
Time to switching (days), median (IQR)	76 (34–122)	79 (34–123)	74 (34–121)	
First switch, *n* (%)				
Apixaban	435 (16.4)	224 (17.9)	211 (15.1)	0.0076
Rivaroxaban	936 (35.4)	456 (36.4)	480 (34.5)	
VKA	561 (21.2)	263 (21.0)	298 (21.4)	
Unfractionated heparin	245 (9.3)	89 (7.1)	156 (11.2)	
Other	468 (17.7)	220 (17.6)	248 (17.8)	

^a^ Chi-square test for categorical variables and ANOVA for continuous variables. Abbreviations: AC, anticoagulant; IQR, interquartile range; LMWH, low molecular weight heparin; SD, standard deviation; VKA, vitamin K antagonist.

**Table 4:** Clinical outcomes at 6 months.

	LMWH		
Clinical Outcomes, 6 Months	All (n = 31,771)	AC-Naïve (n = 14,107)	AC-Experienced (n = 17,664)
VTE recurrence, *n* (%)	1256 (4.0)	523 (3.7)	733 (4.1)
VTE recurrence, crude rate per 100 PM (95% CI)	0.90 (0.86; 0.95)	0.85 (0.78; 0.92)	0.95 (0.88; 1.02)
VTE recurrence: PE (with or without DVT), *n* (%)	700 (2.2)	295 (2.1)	405 (2.3)
VTE recurrence: PE (with or without DVT), crude rate per 100 PM (95% CI)	0.50 (0.47; 0.54)	0.48 (0.43; 0.53)	0.53 (0.48; 0.58)
VTE recurrence: DVT without PE, *n* (%)	556 (1.8)	228 (1.6)	328 (1.9)
VTE recurrence: DVT without PE, crude rate per 100 PM (95% CI)	0.40 (0.37; 0.43)	0.37 (0.32; 0.42)	0.43 (0.38; 0.47)
Overall bleeding leading to hospitalization, principal diagnosis, *n* (%)	1124 (3.5)	441 (3.1)	683 (3.9)
Overall bleeding leading to hospitalization, crude rate per 100 PM (95% CI)	0.81 (0.76; 0.86)	0.71 (0.65; 0.78)	0.89 (0.82; 0.95)
Intracranial bleeding, principal diagnosis at 6 months *n* (%)	130 (0.4)	59 (0.4)	71 (0.4)
Intracranial bleeding, crude rate per 100 PM (95% CI)	0.09 (0.08; 0.11)	0.10 (0.07; 0.12)	0.09 (0.07; 0.12)
Gastrointestinal bleeding, principal diagnosis, *n* (%)	432 (1.4)	156 (1.1)	276 (1.6)
Gastrointestinal bleeding, crude rate per 100 PM (95% CI)	0.31 (0.28; 0.34)	0.25 (0.22; 0.29)	0.36 (0.32; 0.4)
Bleeding from other sites, principal diagnosis, *n* (%) ^a^	570 (1.8)	227 (1.6)	343 (1.9)
Bleeding from other sites, principal diagnosis, crude rate per 100 PM (95% CI)	0.41 (0.38; 0.45)	0.37 (0.32; 0.42)	0.44 (0.4; 0.49)
CTEPH, *n* (%)	58 (0.2)	18 (0.1)	40 (0.2)
CTEPH, crude rate per 100 PM (95% CI)	0.04 (0.03; 0.05)	0.03 (0.02; 0.05)	0.05 (0.04; 0.07)
All-cause death, *n* (%)	10,383 (32.7)	4465 (31.7)	5918 (33.5)
All-cause death, crude rate per 100 PM (95% CI)	7.47 (7.33; 7.61)	7.22 (7.02; 7.43)	7.67 (7.49; 7.86)

^a^ Other bleeding sites include: uterine and vaginal, intraocular, otorrhagia, pericardial, respiratory, and intra-articular. Abbreviations: AC, anticoagulant; CI, confidence interval; CTEPH, chronic thromboembolic pulmonary hypertension; DVT, deep vein thrombosis; LMWH, low molecular weight heparin; PE, pulmonary embolism; PM, person-months; VTE, venous thromboembolism.

**Table 5:** Clinical outcomes at 12 months.

	LMWH		
Clinical Outcomes, 12 Months	All (n = 31,771)	AC-Naïve (n = 14,107)	AC-Experienced (n = 17,664)
VTE recurrence, *n* (%)	1546 (4.9)	652 (4.6)	894 (5.1)
VTE recurrence, crude rate per 100 PM (95% CI)	0.71 (0.67; 0.74)	0.67 (0.62; 0.72)	0.74 (0.69; 0.78)
VTE recurrence: PE (with or without DVT), *n* (%)	887 (2.8)	378 (2.7)	509 (2.9)
VTE recurrence: PE (with or without DVT), crude rate per 100 PM (95% CI)	0.40 (0.38; 0.43)	0.39 (0.35; 0.43)	0.42 (0.38; 0.46)
VTE recurrence: DVT without PE, *n* (%)	659 (2.1)	274 (1.9)	385 (2.2)
VTE recurrence: DVT without PE, crude rate per 100 PM (95% CI)	0.30 (0.28; 0.32)	0.28 (0.25; 0.32)	0.32 (0.29; 0.35)
Overall bleeding leading to hospitalization, principal diagnosis, *n* (%)	1438 (4.5)	566 (4.0)	872 (4.9)
Overall bleeding leading to hospitalization, principal diagnosis, crude rate per 100 PM (95% CI)	0.66 (0.62; 0.69)	0.58 (0.53; 0.63)	0.72 (0.67; 0.77)
Intracranial bleeding, principal diagnosis, *n* (%)	176 (0.6)	85 (0.6)	91 (0.5)
Intracranial bleeding, principal diagnosis, crude rate per 100 PM (95% CI)	0.08 (0.07; 0.09)	0.09 (0.07; 0.11)	0.07 (0.06; 0.09)
Gastrointestinal bleeding, principal diagnosis, *n* (%)	550 (1.7)	197 (1.4)	353 (2.0)
Gastrointestinal bleeding, principal diagnosis, crude rate per 100 PM (95% CI)	0.25 (0.23; 0.27)	0.2 (0.18; 0.23)	0.29 (0.26; 0.32)
Bleeding from other sites, principal diagnosis, *n* (%) ^a^	722 (2.3)	286 (2.0)	436 (2.5)
Bleeding from other sites, principal diagnosis, crude rate per 100 PM (95% CI)	0.33 (0.31; 0.35)	0.29 (0.26; 0.33)	0.36 (0.33; 0.39)
CTEPH, *n* (%)	74 (0.2)	26 (0.2)	48 (0.3)
CTEPH, crude rate per 100 PM (95% CI)	0.03 (0.03; 0.04)	0.03 (0.02; 0.04)	0.04 (0.03; 0.05)
All-cause death, *n* (%)	14,124 (44.5)	6034 (42.8)	8090 (45.8)
All-cause death, crude rate per 100 PM (95% CI)	6.44 (6.34; 6.55)	6.19 (6.04; 6.34)	6.65 (6.51; 6.79)

^a^ Other bleeding sites include: uterine and vaginal, intraocular, otorrhagia, pericardial, respiratory, and intra-articular. Abbreviations: AC, anticoagulant; CI, confidence interval; CTEPH, chronic thromboembolic pulmonary hypertension; DVT, deep vein thrombosis; LMWH, low molecular weight heparin; PE, pulmonary embolism; PM, person-months; VTE, venous thromboembolism.

## Data Availability

The patient-level data used for this study are not publicly available due to privacy restrictions per French law and the General Data Protection Regulation (GDPR).

## Supplementary Materials

The following supporting information can be downloaded at: https://www.mdpi.com/article/10.3390/cancers15113011/s1, Figure S1: Clinical outcomes in patients treated with LMWH at 6 months follow-up. Data are event rates (95% CI) per 100 person-months. Abbreviations: CI, confidence interval; DVT, deep vein thrombosis; GI, gastrointestinal; ICH, intracerebral hemorrhage; LMWH, low molecular weight heparin; PE, pulmonary embolism; VTE, venous thromboembolism; Table S1: Cancer-related treatments and procedures in LMWH-treated patients with VTE; Table S2: Patient disposition according to follow-up time; Table S3: Clinical characteristics of patients in subgroups 1 and 2; Table S4: Clinical outcomes at 6 months for patients in subgroups 1 and 2; Table S5: Clinical outcomes at 12 months for patients in subgroups 1 and 2.

## References

[1] Song A.B., Rosovsky R.P., Connors J.M., Al-Samkari H. (2019). Direct oral anticoagulants for treatment and prevention of venous thromboembolism in cancer patients. Vasc. Health Risk Manag..

[2] Trinh V.Q., Karakiewicz P.I., Sammon J., Sun M., Sukumar S., Gervais M.-K., Shariat S.F., Tian Z., Kim S.P., Kowalczyk K.J. (2014). Venous Thromboembolism After Major Cancer Surgery: Temporal Trends and Patterns of Care. JAMA Surg..

[3] Barbosa M. (2014). What is the Best Treatment for a Cancer Patient with Thrombosis?. Clin. Med. Insights Oncol..

[4] Gimbel I.A., Mulder F.I., Bosch F.T.M., Freund J.E., Guman N., van Es N., Kamphuisen P.W., Büller H.R., Middeldorp S. (2021). Pulmonary embolism at autopsy in cancer patients. J. Thromb. Haemost..

[5] Laporte S., Mismetti P., Décousus H., Uresandi F., Otero R., Lobo J.L., Monreal M. (2008). Clinical Predictors for Fatal Pulmonary Embolism in 15 520 Patients With Venous Thromboembolism. Circulation.

[6] Chew H.K., Wun T., Harvey D., Zhou H., White R.H. (2006). Incidence of venous thromboembolism and its effect on survival among patients with common cancers. Arch. Intern. Med..

[7] Khorana A.A., Francis C.W., Culakova E., Fisher R.I., Kuderer N.M., Lyman G.H. (2006). Thromboembolism in hospitalized neutropenic cancer patients. J. Clin. Oncol..

[8] Prandoni P., Lensing A.W., Cogo A., Cuppini S., Villalta S., Carta M., Cattelan A.M., Polistena P., Bernardi E., Prins M.H. (1996). The long-term clinical course of acute deep venous thrombosis. Ann. Intern. Med..

[9] Xiong W. (2021). Current status of treatment of cancer-associated venous thromboembolism. Thromb. J..

[10] Lyman G.H., Carrier M., Ay C., Di Nisio M., Hicks L.K., Khorana A.A., Leavitt A.D., Lee A.Y.Y., Macbeth F., Morgan R.L. (2021). American Society of Hematology 2021 guidelines for management of venous thromboembolism: Prevention and treatment in patients with cancer. Blood Adv..

[11] Nishioka J., Goodin S. (2007). Low-molecular-weight heparin in cancer-associated thrombosis: Treatment, secondary prevention, and survival. J. Oncol. Pharm. Pract..

[12] Key N.S., Khorana A.A., Kuderer N.M., Bohlke K., Lee A.Y.Y., Arcelus J.I., Wong S.L., Balaban E.P., Flowers C.R., Francis C.W. (2020). Venous Thromboembolism Prophylaxis and Treatment in Patients With Cancer: ASCO Clinical Practice Guideline Update. J. Clin. Oncol..

[13] Streiff M.B., Holmstrom B., Angelini D., Ashrani A., Bockenstedt P.L., Chesney C., Fanikos J., Fenninger R.B., Fogerty A.E., Gao S. (2018). NCCN Guidelines Insights: Cancer-Associated Venous Thromboembolic Disease, Version 2.2018. J. Natl. Compr. Cancer Netw..

[14] Bertoletti L., Gusto G., Khachatryan A., Quignot N., Chaves J., Moniot A., Mokgokong R. (2022). Effectiveness and Safety of Oral Anticoagulants in the Treatment of Acute Venous Thromboembolism: A Nationwide Comparative Cohort Study in France. Thromb. Haemost..

[15] Catella-Chatron J., Merah A., De Magalhaes E., Moulin N., Accassat S., Duvillard C., Mismetti P., Bertoletti L. (2019). Chronic thromboembolic pulmonary hypertension suspicion after pulmonary embolism in cancer patients. Respir. Med. Res..

[16] Lyon A.R., López-Fernández T., Couch L.S., Asteggiano R., Aznar M.C., Bergler-Klein J., Boriani G., Cardinale D., Cordoba R., Cosyns B. (2022). 2022 ESC Guidelines on cardio-oncology developed in collaboration with the European Hematology Association (EHA), the European Society for Therapeutic Radiology and Oncology (ESTRO) and the International Cardio-Oncology Society (IC-OS): Developed by the task force on cardio-oncology of the European Society of Cardiology (ESC). Eur. Heart J..

[17] Khorana A.A., Kuderer N.M., Culakova E., Lyman G.H., Francis C.W. (2008). Development and validation of a predictive model for chemotherapy-associated thrombosis. Blood.

[18] Petit B., Soudet S., Poenou G., Zarrat E., Machuron T., Accassat S., Plaisance L., Helfer H., Mismetti V., Le Hello C. (2022). PO-41: Cancer-associated thrombosis: How many patients seen in clinical practice would be eligible to a randomized controlled trial?. Thromb. Res..

[19] Girard P., Laporte S., Chapelle C., Falvo N., Falchero L., Cloarec N., Monnet I., Burnod A., Tomasini P., Boulon C. (2021). Failure of the Ottawa Score to Predict the Risk of Recurrent Venous Thromboembolism in Cancer Patients: The Prospective PREDICARE Cohort Study. Thromb. Haemost..

[20] Jara-Palomares L., Solier-Lopez A., Elias-Hernandez T., Asensio-Cruz M., Blasco-Esquivias I., Marin-Barrera L., de la Borbolla-Artacho M.R., Praena-Fernandez J.M., Montero-Romero E., Navarro-Herrero S. (2017). Tinzaparin in cancer associated thrombosis beyond 6 months: TiCAT study. Thromb. Res..

[21] Cohen A.T., Katholing A., Rietbrock S., Bamber L., Martinez C. (2017). Epidemiology of first and recurrent venous thromboembolism in patients with active cancer. A population-based cohort study. Thromb. Haemost..

[22] Mahé I., Plaisance L., Chapelle C., Laporte S., Planquette B., Bertoletti L., Couturaud F., Falvo N., Falchero L., Mahé I. (2020). Long-Term Treatment of Cancer-Associated Thrombosis (CAT) Beyond 6 Months in the Medical Practice: USCAT, a 432-Patient Retrospective Non-Interventional Study. Cancers.

[23] Taylor H.S., Giudice L.C., Lessey B.A., Abrao M.S., Kotarski J., Archer D.F., Diamond M.P., Surrey E., Johnson N.P., Watts N.B. (2017). Treatment of Endometriosis-Associated Pain with Elagolix, an Oral GnRH Antagonist. N. Engl. J. Med..

[24] Klok F.A., Ageno W., Ay C., Bäck M., Barco S., Bertoletti L., Becattini C., Carlsen J., Delcroix M., van Es N. (2022). Optimal follow-up after acute pulmonary embolism: A position paper of the European Society of Cardiology Working Group on Pulmonary Circulation and Right Ventricular Function, in collaboration with the European Society of Cardiology Working Group on Atherosclerosis and Vascular Biology, endorsed by the European Respiratory Society. Eur. Heart J..

[25] Stevens S.M., Woller S.C., Kreuziger L.B., Bounameaux H., Doerschug K., Geersing G.J., Huisman M.V., Kearon C., King C.S., Knighton A.J. (2021). Antithrombotic Therapy for VTE Disease: Second Update of the CHEST Guideline and Expert Panel Report. Chest.

[26] Planquette B., Bertoletti L., Charles-Nelson A., Laporte S., Grange C., Mahé I., Pernod G., Elias A., Couturaud F., Falvo N. (2022). Rivaroxaban vs Dalteparin in Cancer-Associated Thromboembolism: A Randomized Trial. Chest.

[27] McBane R.D., Wysokinski W.E., Le-Rademacher J.G., Zemla T., Ashrani A., Tafur A., Perepu U., Anderson D., Gundabolu K., Kuzma C. (2020). Apixaban and dalteparin in active malignancy-associated venous thromboembolism: The ADAM VTE trial. J. Thromb. Haemost..

[28] Agnelli G., Becattini C., Meyer G., Muñoz A., Huisman M.V., Connors J.M., Cohen A., Bauersachs R., Brenner B., Torbicki A. (2020). Apixaban for the Treatment of Venous Thromboembolism Associated with Cancer. N. Engl. J. Med..

[29] Cohen A., Keshishian A., Lee T., Wygant G., Rosenblatt L., Hlavacek P., Mardekian J., Wiederkehr D., Sah J., Luo X. (2021). Effectiveness and Safety of Apixaban, Low-Molecular-Weight Heparin, and Warfarin among Venous Thromboembolism Patients with Active Cancer: A U.S. Claims Data Analysis. Thromb. Haemost..

[30] Raskob G.E., van Es N., Verhamme P., Carrier M., Di Nisio M., Garcia D., Grosso M.A., Kakkar A.K., Kovacs M.J., Mercuri M.F. (2017). Edoxaban for the Treatment of Cancer-Associated Venous Thromboembolism. N. Engl. J. Med..

[31] Young A.M., Marshall A., Thirlwall J., Chapman O., Lokare A., Hill C., Hale D., Dunn J.A., Lyman G.H., Hutchinson C. (2018). Comparison of an Oral Factor Xa Inhibitor With Low Molecular Weight Heparin in Patients With Cancer With Venous Thromboembolism: Results of a Randomized Trial (SELECT-D). J. Clin. Oncol..

[32] Thapa N., Shatzel J., Deloughery T.G., Olson S.R. (2019). Direct oral anticoagulants in gastrointestinal malignancies: Is the convenience worth the risk?. J. Gastrointest. Oncol..

[33] Khorana A.A., Noble S., Lee A.Y.Y., Soff G., Meyer G., O’Connell C., Carrier M. (2018). Role of direct oral anticoagulants in the treatment of cancer-associated venous thromboembolism: Guidance from the SSC of the ISTH. J. Thromb. Haemost..

[34] Farge D., Frere C., Connors J.M., Ay C., Khorana A.A., Munoz A., Brenner B., Kakkar A., Rafii H., Solymoss S. (2019). 2019 international clinical practice guidelines for the treatment and prophylaxis of venous thromboembolism in patients with cancer. Lancet Oncol..

[35] Catella J., Bertoletti L., Moustafa F., Nieto J.A., Valle R., Pedrajas J.M., Villalobos A., Quere I., Sarlon-Bartoli G., Monreal M. (2022). Major gastrointestinal bleeding in patients receiving anticoagulant therapy for venous thromboembolism. Thromb. Res..

[36] Moik F., Colling M., Mahé I., Jara-Palomares L., Pabinger I., Ay C. (2022). Extended anticoagulation treatment for cancer-associated thrombosis—Rates of recurrence and bleeding beyond 6 months: A systematic review. J. Thromb. Haemost..

[37] McBane R.D., Loprinzi C.L., Ashrani A., Lenz C.J., Houghton D., Zemla T., Le-Rademacher J.G., Wysokinski W.E. (2020). Extending venous thromboembolism secondary prevention with apixaban in cancer patients: The EVE trial. Eur. J. Haematol..

[38] Mahé I., Agnelli G., Ay C., Bamias A., Becattini C., Carrier M., Chapelle C., Cohen A.T., Girard P., Huisman M.V. (2022). Extended Anticoagulant Treatment with Full- or Reduced-Dose Apixaban in Patients with Cancer-Associated Venous Thromboembolism: Rationale and Design of the API-CAT Study. Thromb. Haemost..

[39] Noble S. (2019). Venous thromboembolism and palliative care. Clin. Med..

