# Supplementary material for: Low Molecular Weight Heparin Treatment Patterns and Outcomes in Cancer Patients with Acute Venous Thromboembolism: A Nationwide Cohort Study in France
Source: Cancers (Basel). 2023 May 31;15(11):3011. doi: 10.3390/cancers15113011 (PMC10251904; doi:10.3390/cancers15113011)
Supplement: Supplementary file 1 [file cancers-15-03011-s001.zip › cancers-2274758-supplementary.pdf]

Supplementary Materials: Low Molecular Weight Heparin Treatment Patterns and Outcomes in Cancer Patients with Acute Venous Thromboembolism: A Nationwide Cohort Study in France

Laurent Bertoletti, Gaelle Gusto, Nadia Quignot, Artak Khachatryan, Jose Chaves, Audrey Moniot, Ruth Mokgokong and Isabelle Mahé

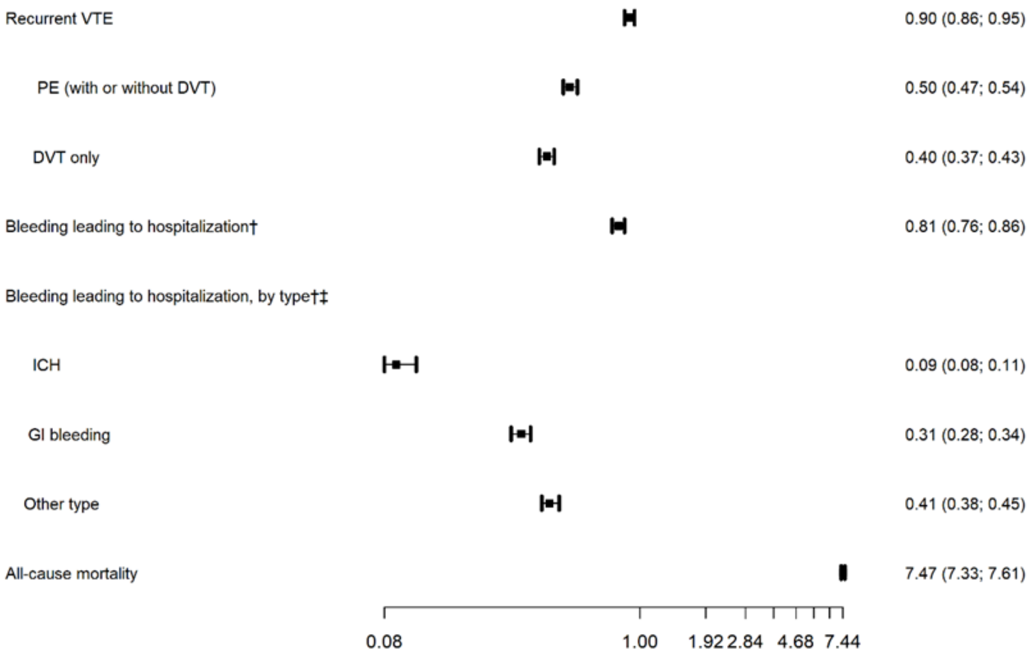

Figure S1. Clinical outcomes in patients treated with LMWH at 6 months follow-up. Data are event rates (95% CI) per 100 person-months. Abbreviations: CI, confidence interval; DVT, deep vein thrombosis; GI, gastrointestinal; ICH, intracerebral hemorrhage; LMWH, low molecular weight heparin; PE, pulmonary embolism; VTE, venous thromboembolism.

Table S1. Cancer-related treatments and procedures in LMWH-treated patients with VTE.

Criteria	n (%)
Any criteria	31,771 (100)
Criterion 1 only	7,535 (23.7)
Criterion 2 only	879 (2.8)
Criterion 3 only	754 (2.4)
Criteria 1 and 2	638 (2.0)
Criteria 1 and 3	955 (3.0)
Criteria 2 and 3	613 (1.9)
Criteria 1, 2, and 3	20,397 (64.2)
Cancer-related ICD-10 code (active cancer)	30,892 (97.2)

Criterion 1: ICD-10 discharge diagnosis for cancer in the hospital database as the principal diagnosis or ≥2 associated diagnoses or ALD registration within 6 months before or 30 days after the index VTE event. Criterion 2: Record of ≥1 of the following cancer-related treatments and procedures within 6 months before or 30 days after the index VTE event: chemotherapy treatment for cancer, follow-up exam or convalescence for chemotherapy, radiotherapy for cancer. Criterion 3: Record of immunotherapy or hormonotherapy or any chemotherapy within 6 months before or 30 days after the index VTE event AND record of an ICD-10 code for cancer (any diagnosis for a hospital stay within 6 months before or 30 days after the index VTE event or ALD registration within 12 months

before or 30 days after the index VTE event). Abbreviations: ALD, *affections de longue durée* (long-term illness/disease); ICD, International Classification of Diseases; LMWH, low molecular weight heparin; VTE, venous thromboembolism.

Table S2. Patient disposition according to follow-up time.

	**Follow-up period**			
	**0–3 months**	**>3–6 months**	**>6–12 months**	**>12 months**
All patients (N=31,771), n (%)				
Included at start of follow-up period	31,771 (100.0)	24,869 (78.3)	20,882 (65.7)	14,751 (46.4)
Died during follow-up	6,827 (21.5)	3,909 (12.3)	4,270 (13.4)	5,544 (17.4)
Alive at end of follow-up	24,944 (78.5)	20,960 (66.0)	16,612 (52.3)	9,207 (29.0)
Lost to follow-up	75 (0.2)	78 (0.2)	1861 (5.9)	
Stopped or switched index treatment[a]	6,758 (27.1)	3,776 (18.0)	3,959 (23.8)	2,439 (16.5)
AC-naïve (N=14,107), n (%)				
Included at start of follow-up period	14,107 (100.0)	11,128 (78.9)	9,442 (66.9)	6,723 (47.7)
Died during follow-up	2,954 (20.9)	1,653 (11.7)	1,809 (12.8)	2,265 (16.1)
Alive at end of follow-up	11,153 (79.1)	9,475 (67.2)	7,633 (54.1)	4,458 (31.6)
Lost to follow-up	25 (0.2)	33 (0.2)	910 (6.5)	
Stopped or switched index treatment[a]	2,982 (26.7)	1,792 (18.9)	1,893 (24.8)	1,114 (16.6)
AC-experienced (N=17,664), n (%)				
Included at start of follow-up period	17,664 (100.0)	13,741 (77.8)	11,440 (64.8)	8,028 (45.4)
Died during follow-up	3,873 (21.9)	2,256 (12.8)	2,461 (13.9)	3,279 (18.6)
Alive at end of follow-up	13,791 (78.1)	11,485 (65.0)	8,979 (50.8)	
Lost to follow-up	50 (0.3)	45 (0.3)	951 (5.4)	
Stopped or switched index treatment[a]	3,776 (27.4)	1,984 (17.3)	2,066 (23.0)	1,325 (16.5)

[a]Among patients who were still alive at the end of the follow-up period. Abbreviations: AC, anticoagulant.

Table S3. Clinical characteristics of patients in subgroups 1 and 2.

	**Subgroup 1 (N=25,256)**	**Subgroup 2 (N=3,805)**	**P-value (subgroup 2 vs. subgroup 1)[a]**
Age at index date (years), mean (SD)	66.1 (13.2)	67.2 (12.9)	<0.0001
Male, n (%)	12,996 (51.5)	1,850 (48.6)	0.004
Deprivation index Q4, n (%)	9,102 (36.0)	1,304 (34.3)	0.056
Deprivation index Q5, n (%)	1,301 (5.2)	163 (4.3)	0.016
Type of VTE, n (%)			
DVT	10,740 (42.5)	1,217 (32.0)	<0.0001
PE (with or without DVT)	14,516 (57.5)	2,588 (68.0)	
Risk of incident VTE based on cancer type, n (%)[b]			
Very high-risk cancer types: brain, pancreatic, stomach	4,347 (17.2)	317 (8.3)	<0.0001
High-risk cancer types: lung, lymphoma, gynecologic[c], bladder, testicular, renal cell carcinoma	11,638 (46.1)	1,568 (41.2)	
Other cancer types	8,966 (35.5)	1,786 (46.9)	
Unknown cancer (ICD-10 information missing)	305 (1.2)	134 (3.5)	
Metastatic vs non metastatic disease, n (%)			
Metastatic disease (C77*-C80*)	18,488 (74.9)	1,958 (52.8)	<0.0001
Non-metastatic disease	5,901 (23.9)	1,615 (43.6)	
Unknown (ICD-10 information missing)	305 (1.2)	134 (3.6)	
Baseline comorbidities (assessed 24 months prior)			

CCI, mean (SD)	6.7 (3.2)	5.3 (3.2)	<0.0001
CCI 2, n (%)	4,713 (18.7)	1,186 (31.2)	<0.0001
CCI 3, n (%)	2,266 (9.0)	502 (13.2)	
CCI 4 or more, n (%)	18,277 (72.4)	2,117 (55.6)	
Baseline bleed, all diagnoses, n (%)	3,252 (12.9)	429 (11.3)	0.006
Baseline bleed, principal diagnosis only, n (%)	1,070 (4.2)	135 (3.5)	0.06
Comorbidities, n (%)			
Hypertension	8,761 (34.7)	1,478 (38.8)	<0.0001
Anemia	7,955 (31.5)	947 (24.9)	<0.0001
Diabetes	4,205 (16.6)	578 (15.2)	0.066
Pulmonary disease	2,977 (11.8)	411 (10.8)	0.043
Obesity	2,709 (10.7)	623 (16.4)	<0.0001
Cerebrovascular disease	1,155 (4.6)	184 (4.8)	0.71
Moderate to severe renal disease	1,075 (4.3)	167 (4.4)	0.75
History of falls	525 (2.1)	78 (2.0)	0.72
LMWH treatment duration (months), mean (SD)	7.04 (9.1)	7.78 (9.9)	<0.0001

[a] Chi-square test for categorical variables and ANOVA for continuous variables. [b] Based on the Khorana score. [c] Gynecologic cancers included malignant neoplasms of the vulva, vagina, cervix uteri, corpus uteri, uterus part unspecified, ovary, placenta, and other and unspecified female genital organs. Abbreviations: CCI, Charlson Comorbidity Index; DVT, deep vein thrombosis; ICD, International Classification of Diseases; LMWH, low molecular weight heparin; PE, pulmonary embolism; Q, quintile; SD, standard deviation; VTE, venous thromboembolism.

Table S4. Clinical outcomes at 6 months for patients in subgroups 1 and 2.

**Clinical outcomes, 6 months**	**Subgroup 1 (N=25,256)**	**Subgroup 2 (N=3,805)**
VTE recurrence, n (%)	1,038 (4.1)	131 (3.4)
VTE recurrence, crude rate per 100 PM (95% CI)	0.90 (0.85; 0.96)	0.85 (0.72; 1.01)
VTE recurrence: PE (with or without DVT), n (%)	589 (2.3)	62 (1.6)
VTE recurrence: PE (with or without DVT), crude rate per 100 PM (95% CI)	0.51 (0.47; 0.55)	0.4 (0.31; 0.52)
VTE recurrence: DVT only, n (%)	449 (1.8)	69 (1.8)
VTE recurrence: DVT only, crude rate per 100 PM (95% CI)	0.39 (0.36; 0.43)	0.45 (0.35; 0.57)
Overall bleeding, principal diagnosis, n (%)	1,008 (4.0)	66 (1.7)
Overall bleeding, principal diagnosis, crude rate per 100 PM (95% CI)	0.87 (0.82; 0.93)	0.43 (0.34; 0.55)
Intracranial bleeding, principal diagnosis, n (%)	123 (0.5)	<10
Intracranial bleeding, principal diagnosis, crude rate per 100 PM (95% CI)	0.11 (0.09; 0.13)	0.03 (0.01; 0.08)
Gastrointestinal bleeding, principal diagnosis, n (%)	395 (1.6)	19 (0.5)
Gastrointestinal bleeding, principal diagnosis, crude rate per 100 PM (95% CI)	0.34 (0.31; 0.38)	0.12 (0.08; 0.19)
Bleeding from other sites, principal diagnosis, n (%)[a]	498 (2.0)	42 (1.1)
Bleeding from other sites, principal diagnosis, crude rate per 100 PM (95% CI)	0.43 (0.40; 0.47)	0.27 (0.20; 0.37)
CTEPH, n (%)	48 (0.2)	<10
CTEPH, crude rate per 100 PM (95% CI)	0.04 (0.03; 0.06)	0.05 (0.02; 0.10)
All-cause death, n (%)[b]	10,383 (41.1)	-
All-cause death, crude rate per 100 PM (95% CI)	9.01 (8.84; 9.17)	-

[a] Other bleeding sites include: uterine and vaginal, intraocular, otorrhagia, pericardial, respiratory, and intra-articular. [b] Data were censored at treatment switching, so data on death was not collected for subgroup 2. Abbreviations: AC, anticoagulant; CI, confidence interval; CTEPH, chronic thromboembolic pulmonary hypertension; DVT, deep vein thrombosis; PE, pulmonary embolism; PM, person-months; VTE, venous thromboembolism.

Table S5. Clinical outcomes at 12 months for patients in subgroups 1 and 2.

Clinical outcomes, 12 months	Subgroup 1 (N=25,256)	Subgroup 2 (N=3,805)
VTE recurrence, n (%)	1,264 (5.0)	178 (4.7)
VTE recurrence, crude rate per 100 PM (95% CI)	0.68 (0.64; 0.71)	0.84 (0.73; 0.98)
VTE recurrence: PE (with or without DVT), n (%)	740 (2.9)	89 (2.3)
VTE recurrence: PE (with or without DVT), crude rate per 100 PM (95% CI)	0.40 (0.37; 0.43)	0.42 (0.34; 0.52)
VTE recurrence: DVT only, n (%)	524 (2.1)	89 (2.3)
VTE recurrence: DVT only, crude rate per 100 PM (95% CI)	0.28 (0.26; 0.31)	0.42 (0.34; 0.52)
Overall bleeding, principal diagnosis, n (%)	1,301 (5.2)	81 (2.1)
Overall bleeding, principal diagnosis, crude rate per 100 PM (95% CI)	0.70 (0.66; 0.74)	0.38 (0.31; 0.48)
Intracranial bleeding, principal diagnosis, n (%)	167 (0.7)	<10
Intracranial bleeding, principal diagnosis, crude rate per 100 PM (95% CI)	0.09 (0.08; 0.10)	0.03 (0.01; 0.06)
Gastrointestinal bleeding, principal diagnosis, n (%)	506 (2.0)	24 (0.6)
Gastrointestinal bleeding, principal diagnosis, crude rate per 100 PM (95% CI)	0.27 (0.25; 0.30)	0.11 (0.08; 0.17)
Bleeding from other sites, principal diagnosis, n (%)[a]	638 (2.5)	51 (1.3)
Bleeding from other sites, principal diagnosis, crude rate per 100 PM (95% CI)	0.34 (0.32; 0.37)	0.24 (0.18; 0.32)
CTEPH, n (%)	60 (0.2)	<10
CTEPH, crude rate per 100 PM (95% CI)	0.03 (0.02; 0.04)	0.04 (0.02; 0.08)
All-cause death, n (%)[b]	14,124 (55.9)	-
All-cause death, crude rate per 100 PM (95% CI)	7.56 (7.44; 7.68)	-

[a] Other bleeding sites include: uterine and vaginal, intraocular, otorrhagia, pericardial, respiratory, and intra-articular. [b] Data was censored at treatment switching, so data on death was not collected for subgroup 2. Abbreviations: AC, anticoagulant; CI, confidence interval; CTEPH, chronic thromboembolic pulmonary hypertension; DVT, deep vein thrombosis; PE, pulmonary embolism; PM, person-months; VTE, venous thromboembolism.
